# The Pathology of Mediastinal Tumours, with Special Reference to Diagnosis

**Published:** 1892-09

**Authors:** 


*The Pathology of Mediastinal Tumours, with special reference to Diagnosis.* By JOHN LINDSAY STEVEN, M.D. Pp. 100. London: H. K. LEWIS. 1892.

This volume is founded on a short course of post-graduate lectures, delivered in Glasgow in 1890. It contains a short outline of the literature of the subject, and then gives illustrative cases of the principal groups of mediastinal growths. In considering the question of diagnosis, the author refers especially to the following useful points :

1. The development of fulness and nodular or glandular projections beneath the clavicles and in the neck, such as are specially characteristic of lymph-sarcoma.
2. The development of secondary nodules elsewhere.
3. Spasmodic asthma, and paralysis of the vocal cords.
4. Local œdema and local venous varicosity.

"Not only should we be able to diagnose clinically the presence of a solid tumour within the chest, but we should also be able to arrive at a tolerably clear idea of the kind of solid tumour with which we are dealing." The book will materially aid in a solution of this problem.

